# Supplementary material for: Machine learning-based prediction of diabetic retinopathy from pupillary abnormalities in a South Indian population
Source: PLoS One. 2026 Jan 22;21(1):e0340802. doi: 10.1371/journal.pone.0340802 (PMC12826491; doi:10.1371/journal.pone.0340802)
Supplement: S1 Table — (DOCX) [file pone.0340802.s001.docx]

**Table S1: Comparative performance metrics of Machine Learning Models across Training, Test, and Validation Sets without using SMOTE**

| **Models** | **Training(N=104)** | | **Validation(N=19)** | | **Test (N=22)** | |
| --- | --- | --- | --- | --- | --- | --- |
|  | **Accuracy** | **AUC** | **Accuracy** | **AUC** | **Accuracy** | **AUC** |
| ANN | 0.89 | 0.76 | 0.76 | 0.79 | 0.54 | 0.59 |
| KNN | 0.73 | 0.70 | 0.58 | 0.61 | 0.64 | 0.56 |
| LR | 0.73 | 0.50 | 0.63 | 0.50 | 0.63 | 0.50 |
| SVM | 0.82 | 0.81 | 0.53 | 0.32 | 0.54 | 0.53 |
| NB | 0.69 | 0.7 | 0.52 | 0.56 | 0.63 | 0.49 |
| RF | 0.87 | 0.99 | 0.58 | 0.44 | 0.64 | 0.54 |
| DT | 0.88 | 0.95 | 0.52 | 0.50 | 0.50 | 0.51 |
