# Supplementary material for: Machine learning-based prediction of diabetic retinopathy from pupillary abnormalities in a South Indian population
Source: PLoS One. 2026 Jan 22;21(1):e0340802. doi: 10.1371/journal.pone.0340802 (PMC12826491; doi:10.1371/journal.pone.0340802)
Supplement: S2 Table — (DOCX) [file pone.0340802.s002.docx]

**Table S2: Effect of Hyperparameter Tuning on Validation Accuracy of the ANN Model**

| **Learning rate** | **No. of neurons** | **Dropout rate** | **Batch size** | **Val accuracy** |
| --- | --- | --- | --- | --- |
| 0.005 | [128,64] | 0.3 | 8 | 0.833 |
| 0.005 | [64,32] | 0 | 8 | 0.854 |
| 0.001 | [128,64] | 0 | 16 | 0.724 |
| 0.005 | [256,128] | 0 | 8 | 0.867 |
| 0.001 | [128,64] | 0 | 8 | 0.793 |
