# Supplementary material for: Machine learning-based prediction of diabetic retinopathy from pupillary abnormalities in a South Indian population
Source: PLoS One. 2026 Jan 22;21(1):e0340802. doi: 10.1371/journal.pone.0340802 (PMC12826491; doi:10.1371/journal.pone.0340802)
Supplement: Attachment — Submitted filename: Response to reviewers.docx [file pone.0340802.s004.docx]

**Response to Reviewers**

**Journal:** PLOS ONE

**Manuscript Number**: PONE-D-25-25752

**Title:** Machine Learning-Based Prediction of Diabetic Retinopathy from Pupillary Abnormalities in a South Indian Population

**Author’s:** Janani Surya R, Tamilselvi S, Maitreyee Roy, Sivaraj chinnasamy, Rajiv Raman, Suchetha M, Ganesh Rajendran

**Associate Editor**

| **Sl. No** | **Associate Editor Comments and Authors’ Response** | **Section & Page No** |
| --- | --- | --- |
| 1. | **Editor’s comment:** Novel approach using pupillometry is interesting, but dataset is small, imbalanced, and lacks external validation. Risk of overfitting (ANN) not fully addressed. | *Section 2&3, Page No: 4,5,6*  *Line number-129-134*  *Line Number-150-180* |
|  | **Author’s response:** We addressed the imbalance using SMOTE and reduced overfitting through hyperparameter tuning and five-fold cross-validation. Despite the small dataset, our ANN showed strong performance in classifying normal and DR cases. We have included the details about hyperparameter tuning in methodology section. |  |
|  |  |  |
| 2. | **Editor’s comment:** Methods / reproducibility: Key details on model training, cross-validation, and parameter tuning are missing. Data/code not publicly available, which conflicts with PLOS ONE policy. | *Section 3, Page No:5,6*  *Line Number-150-180*  *Data availability: Line number-364-370*  *Code availability: Line number-371-373* |
|  | **Author’s response:** We have updated the methodology section by including the training details, cross-validation and hyper parameter tuning. We have created a GitHub repo to made the code publicly available and dataset would be available on request basis. |  |
| 3. | **Editor’s comment:** Discussion / conclusions: Conclusions are overstated given preliminary data. Limitations and comparison with established retinal imaging methods should be expanded. | *Section 5, Page No:9*  *Line number-300-306*  *Section 5, Page No:10*  *Line number-307-340*  *Section 5, Page No:11*  *Line number-341-355* |
|  | **Author’s response:** We appreciate the reviewer’s suggestion. We have revised the Discussion and Conclusion sections to provide a more balanced interpretation of our findings. The revised text acknowledges the preliminary nature of our results and includes a dedicated discussion comparing pupillometry with established retinal imaging approaches. We also explicitly highlight the clinical utility and limitations of pupillary metrics in the broader screening context |  |
| 4. | **Editor’s comment:** Ethics / data availability: Ethics approval is in place, but data availability does not comply with journal requirements; public data/code deposition is expected. | *Data availability: Line number-364-370*  *Code availability: Line number-371-373* |
|  | **Author’s response:** Due to patient-identifiable information, raw pupillometry data cannot be made publicly available. We have created a Git-Hub repo to made the code publicly available and dataset would be available on request basis. All random seeds and hyperparameters used in training are documented within the repository for transparency. |  |

**Reviewer 1**

| **Sl. No** | **Reviewers' Comments and Authors’ Response** | **Section & Page No** |
| --- | --- | --- |
| 1. | **Reviewers' Comment:** The novelty is clear: using pupillary abnormalities instead of fundus images for DR prediction. However, the introduction currently emphasizes prior work on risk scores and fundus-based ML models without sufficiently highlighting how pupillometry could complement or outperform these approaches.  The authors should explicitly emphasize the clinical practicality of pupillometry (speed, cost, portability) compared to fundus photography, particularly in low-resource screening contexts. | *Section 1, Page No:3*  *Line number-85-91* |
|  | **Authors’ Response**: Thank you for this constructive suggestion. In response, we have expanded the Introduction section to better emphasize the clinical practicality and potential advantages of pupillometry over traditional fundus-based approaches, particularly in low-resource or community screening contexts. We have added a new paragraph (Lines 86–92) that highlights the advantages of pupillometry including its portability, cost-effectiveness, and ease of deployment with minimal operator training, making it suitable for frontline DR screening. We also cite recent evidence demonstrating that handheld or automated pupillometry devices can detect early neuro-optic dysfunction even before visible DR changes, underscoring its utility as a triage or adjunct screening tool. Relevant references have been added (27,31,33) to support these claims. |  |
| 2. | **Reviewers' Comment:** The ANN architecture is described, but hyperparameter tuning (layers, neurons, batch size, epochs) seems arbitrary. Were these parameters chosen empirically or optimized systematically? Please clarify.  Tables 3 and 4: It is unclear what input features were used to generate these results (e.g., age, time for one Hippus cycle, BPD, VPC). This needs to be explicitly described in the Methods and clearly displayed in the tables. | *Section 3 and 4, Page no:5&6*  *Line number-150-180*  *Line number-182-200* |
|  | **Author’s Response:** The model architecture and training parameters, including learning rate, number of hidden layers, dropout rate, epochs, and batch size, were optimized through a grid search strategy combined with five-fold stratified cross-validation to ensure balanced and robust evaluation, also the total number of features and the important features selection method and the features used in the model were included in the results section. |  |
| 3. | **Reviewers' Comment:** Table 2 provides cut-offs for pupillary features. While statistically valuable, the clinical interpretability is uncertain. For example, is a BPD cut-off of 4.50 mm meaningful in real-world screening? If possible, the authors should expand the discussion on how pupillometry correlates with DR severity and whether these findings align with, or add predictive value beyond, standard risk factors such as HbA1c and diabetes duration. A comparative discussion would better highlight potential clinical application of the cut-offs. | *Section 5, Page no: 10 Line number -307-329* |
|  | **Author’s response:**  We thank the reviewer for raising this important point. In response, we have expanded the **Discussion** section to address the clinical relevance of the derived cut-offs and how our findings align with prior research on systemic risk factors for DR. Specifically, we highlight the added value of pupillometric markers as potential adjuncts in settings where blood investigations or fundus imaging may not be immediately available. Additionally, we clarify that while the cut-offs such as BPD ≥ 4.50 mm demonstrate statistically significant associations, their clinical translation warrants further validation in prospective and multi-modal screening setups. The revised discussion acknowledges the need for combined models integrating systemic and pupillary parameters to enhance predictive utility. |  |
| 4. | **Reviewers' Comment:** The limitations are understated. The main limitation is that impairments in pupillary dynamics are not disease-specific and must be interpreted in the broader clinical context. As the authors note, changes may reflect diabetic autonomic neuropathy, but pupillary dysfunction can also result from other conditions that affect the vision (e.g., macular degeneration, optic neuropathies). This confounding is critical and must be emphasized.  The dataset is from a single-center, South Indian cohort. The generalizability of findings to other ethnicities and healthcare contexts is limited and should be explicitly acknowledged. | *Section 5, Page no: 10,11 Lines:318-349* |
|  | **Authors’ Response:** We have updates the discussion section including these limitations. |  |
| 5. | **Reviewers' Comment:** The current statement (“Data and code available upon request”) does not meet PLOS ONE data policy. Authors should deposit anonymized data and analysis code in a public repository (e.g., Dryad, Figshare, GitHub). Without this, reproducibility and transparency are limited. | *Data availability: Line number-364-370*  *Code availability: Line number-371-373* |
|  | **Author's Response:** We have created a Git-Hub repo to made the code publicly available and dataset would be available on request basis. |  |
| 6 | **Reviewers' Comment:**  Figures 3 and 4 are useful but require higher resolution and clearer labelling. Figure legends should be made more informative, with all abbreviations defined, so that readers can interpret the figures without referring back to the text. | *Figure 3 and 4 has been updated* |
|  | **Authors’ Response**: We have updated the figures 3 and 4 |  |

**Reviewer 2**

| **Sl. No** | **Reviewers' Comments and Authors’ Response** | **Section & Page No** |
| --- | --- | --- |
| 1. | **Reviewers' Comment:** The use of both eyes from the same patient without clear patient-level data splitting raises concerns about data independence and potential overestimation of model performance | *Section 2, page No: 4, Line number: 131-134* |
|  | **Authors’ Response**: We appreciate the reviewer’s insightful observation. In the revised manuscript, we have clarified that only **one eye per participant** was included for machine learning model development to ensure statistical independence and avoid inter-eye correlation. |  |
| 2. | **Reviewers' Comment:** Although the authors attempted to address class imbalance using SMOTE, the models were trained on the original imbalanced dataset without providing detailed comparative analysis | *Section 2, page No: 4, Line number: 129-134*  *Table 3 and 5, page no: 18* |
|  | **Authors’ Response:** We used SMOTE to address the class imbalance, and it performed well in improving accuracy. The results with SMOTE are shown in Table 3, while the results for the original imbalanced dataset are presented in Table 5. |  |
| 3. | **Reviewers' Comment:** The ANN architecture is relatively simple (with only 2433 parameters) and trained with no regularization techniques which raises concerns for showcase of overfitting | *Section 3, Page No 5 and 6*  *Line number-150-180* |
|  | **Authors’ Response:** Because of the limited size of the dataset, we trained an ANN model to reduce the risk of overfitting. We also applied various regularization techniques and performed hyperparameter tuning to select the parameters for the ANN model that achieved the best performance. The results are presented in Table 3. |  |
| 4. | **Reviewers' Comment:** The ANN performance is very high (accuracy 93%, AUC score 0.96 on test data), yet the validation AUC drops sharply to 0.57% (which suggest possible problems such as overfitting and should be addressed) | *Figure 4* |
|  | **Authors’ Response:** We retrained the model using hyperparameter tuning, cross-validation, and regularization techniques. The refined model achieved a validation accuracy of 0.87 and an AUC of 0.97, demonstrating improved performance without signs of overfitting. |  |
| 5. | **Reviewers' Comment:** The model was trained on relatively small dataset (405 eyes), and validation set size (n=30) which is insufficient to draw strong conclusions | *We have included the updated results in Table 3, page no: 18* |
|  | **Authors’ Response:** The original dataset contains total 145 samples with 102 in normal category and 43 in DR category we applied smote to increase this sample size to (102 samples in normal category and 102 in DR category). We have performed cross validation and bootstrapping with 95% CI to further draw strong conclusions. |  |
| 6. | **Reviewers' Comment:** Standard metrics are reported without consideration of confidence intervals or statistical significance of the results | *Abstract, Page No: 1, Line number: 29-31* |
|  | **Authors’ Response:** We have added 95% CI for the test results. |  |
| 7. | **Reviewers' Comment:**  Additionally, neither the code repository nor the dataset is publicly available, raising concerns about the study’s reproducibility. If data access is restricted, a clear justification should be provided, as stating 'available upon request' is insufficient under standard open data policies (see instructions on how to report this). | *Data availability: Line number-364-370*  *Code availability: Line number-371-373* |
|  | **Authors’ Response:** We have created a Git-Hub repo to made the code publicly available and dataset would be available on request basis. |  |
