# Supplementary material for: Machine learning-based prediction of diabetic retinopathy from pupillary abnormalities in a South Indian population
Source: PLoS One. 2026 Jan 22;21(1):e0340802. doi: 10.1371/journal.pone.0340802 (PMC12826491; doi:10.1371/journal.pone.0340802)
Supplement: Attachment — Submitted filename: Response_to_reviewers_auresp_2.docx [file pone.0340802.s005.docx]

**Response to Reviewers**

**Journal:** PLOS ONE

**Manuscript Number**: PONE-D-25-25752

**Title:** Machine Learning-Based Prediction of Diabetic Retinopathy from Pupillary Abnormalities in a South Indian Population

**Author’s:** Janani Surya R, Tamilselvi S, Maitreyee Roy, Sivaraj chinnasamy, Rajiv Raman, Suchetha M, Ganesh Rajendran

**Reviewer 1**

| **Sl. No** | **Reviewers' Comments and Authors’ Response** | **Section & Page No** |
| --- | --- | --- |
| 1. | **Reviewers' Comment:** Figure 2 is not referenced in the main text and should be cited at the appropriate location. | *Section 3 & page number 6*  *Line number: 172* |
|  | **Authors’ Response:** Thanks for the comment. We have now cited the figure 2 in the model training and implementation section. |  |

**Reviewer 2**

| **Sl. No** | **Reviewers' Comments and Authors’ Response** | **Section & Page No** |
| --- | --- | --- |
| 1. | **Reviewers' Comment:** The revised version state the one eye per participant used, the resulting sample size (145) does not match the number of subjects (244). It remains unclear how the subjects were excluded and how the single eye was selected. | *Section 2, page number: 4*  *Line number: 108-111* |
|  | **Authors’ Response:** For the ML analysis, we selected a subset of 145 participants with complete data and clear labels. Based on DR severity, one eye per participant was included to avoid inter-eye correlation. This subset included 102 no-DR and 43 DR cases. |  |
| 2. | **Reviewers' Comment:** The original submission indicates that SMOTE was tested, but not used in the final analysis. Current version states SMOTE as part of the methodology. It should be clearly stated whether the SMOTE was used pre or post splitting the dataset. Potential data leakage if used in the split. How was SMOTE integrated with cross-validation? | *-* |
|  | **Authors’ Response:**  SMOTE was applied only after splitting the dataset, and never before. The data were first divided into training, validation, and test sets. SMOTE was not applied to the validation or test sets at any stage. For cross-validation, during each fold, SMOTE was applied exclusively to the training portion of that fold, and the validation fold remained untouched. |  |
| 3. | **Reviewers' Comment:** Both SMOTE-based and non-SMOTE analyses are presented. Please state clearly which set of results is used to support the study’s final conclusions. | *Page no: 18, Table 3.* |
|  | **Authors’s Response:** The results of the non-SMOTE analysis were given only for comparative purposes, and results presented in Table 3 supports the study’s conclusion. |  |
| 4. | **Reviewers' Comment:** Hyperparameters were added in the revision, the ANN still includes no regularization (L1/L2 penalties, early stopping) while the dropout is 0. Please explicitly explain what changes to the pipeline led to the improved results (or is it only the effect of the SMOTE?). | *Page 19, Table 6* |
|  | **Authors’ Response:** We have added Table 6 to illustrate how hyperparameter tuning affects the model’s performance. |  |
| 5. | **Reviewers' Comment:** Although 95% confidence intervals are mentioned in the Abstract, they do not appear anywhere in the Results section or in the tables, nor is the bootstrapping methodology described. Please report the full confidence intervals within the Results and explain precisely how they were computed, or remove this claim. | *Bootstrapping-page:5,*  *line number 164-167*  *95% CI- page no:8,*  *line number-245-254*  *Page no:19, Table 4* |
|  | **Authors’ Response:** We have added the 95% confidence intervals for accuracy and AUC for all models in Table 4. The results section has been updated accordingly with these CI values, and the bootstrapping methodology has been explained in detail. |  |
